# Transient Antiphospholipid Syndrome Associated with Primary Cytomegalovirus Infection: A Case Report and Literature Review

**DOI:** 10.1155/2014/271548

**Published:** 2014-12-08

**Authors:** Tsuyoshi Nakayama, Mitsuteru Akahoshi, Kensuke Irino, Yasutaka Kimoto, Yojiro Arinobu, Hiroaki Niiro, Hiroshi Tsukamoto, Takahiko Horiuchi, Koichi Akashi

**Affiliations:** ^1^Department of Clinical Immunology and Rheumatology/Infectious Disease, Kyushu University Hospital, 3-1-1 Maidashi, Higashi-ku, Fukuoka 812-8582, Japan; ^2^Clinical Education Center, Kyushu University Hospital, 3-1-1 Maidashi, Higashi-ku, Fukuoka 812-8582, Japan; ^3^Department of Internal Medicine, Kyushu University Beppu Hospital, 4546 Tsurumibaru, Beppu, Oita 874-0838, Japan

## Abstract

Viral infection is known to induce transient autoimmunity in humans. Acute cytomegalovirus (CMV) infection is implicated in occasional thrombosis formation. We here, for the first time, report a 19-year-old female who had an acute CMV infection, leading to a deep venous thrombosis and a pulmonary embolism along with transient appearance of lupus anticoagulant. The pathological role of antiphospholipid antibodies in CMV-mediated thrombosis is discussed.

## 1. Introduction

Antiphospholipid antibodies (aPL) are circulating antibodies directed against phospholipids. These antibodies are potentially associated with thrombosis and/or pregnancy morbidity and are detected in a variety of conditions, including antiphospholipid syndrome (APS) and systemic lupus erythematosus (SLE). The prevalence of aPL in the form of lupus anticoagulants (LAC) or anticardiolipin antibodies (aCL) is 1–5% of healthy individuals, but the prevalence increases in the elderly and in those with chronic diseases [1]. Viral infection is known to induce transient autoimmunity in humans. The relationship between viral infections and the appearance of aPL has been reported, though infection-induced aPL is generally not associated with thrombotic episodes [2]. Recent studies, however, highlight the risk for either venous or arterial thrombosis in acute cytomegalovirus (CMV) infection in both immunocompromised and immunocompetent patients [3]. Here, we describe a previously healthy 19-year-old woman who developed primary CMV infection complicated by a deep venous thrombosis (DVT), pulmonary embolism (PE), and alveolar hemorrhage along with a transient appearance of LAC. We also review the literature on CMV-induced thrombosis associated with aPL, including our case.

## 2. Case Presentation

A 19-year-old previously healthy Japanese woman was admitted to our hospital with an alveolar hemorrhage, deep vein thrombosis (DVT), and pulmonary embolism (PE) in January 2013. One month before admission, she developed a dry cough, followed by hemosputum, fever, and right-sided chest pain. She was suspected of having pneumonia on the basis of a chest X-ray and was administered antibiotics. However, her symptoms gradually worsened and she was referred to a department of respiratory disease at another hospital. Bronchoscopy revealed alveolar hemorrhage and computed tomography (CT) scans showed DVT and PE. Since she also had additional abnormal findings, such as prolonged dilute Russell viper venom time (dRVVT) and was positive for antinuclear antibodies (ANA, 1 : 320) (Table 1), she was suspected of having SLE-related APS and then transferred to our hospital.

**Figure 1:**
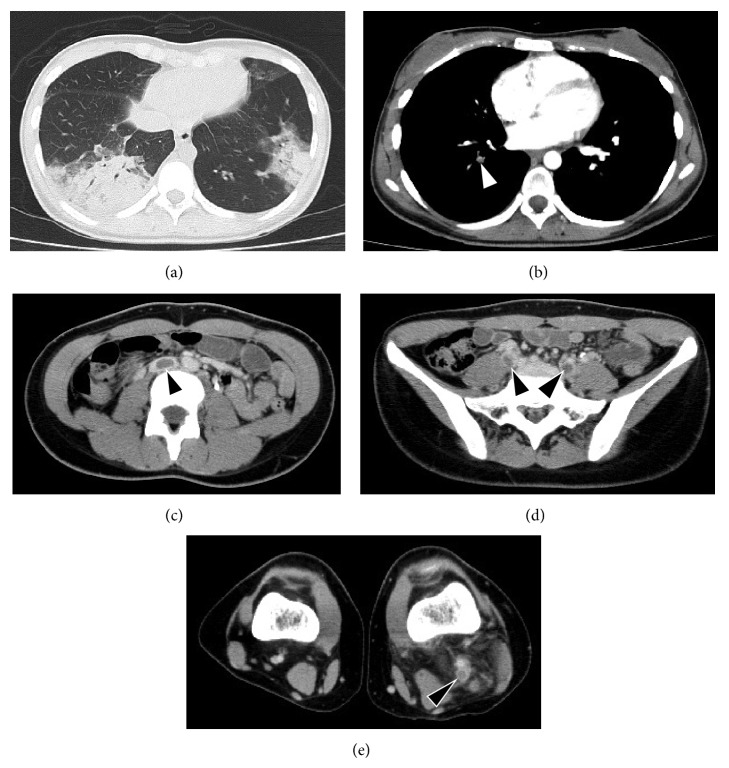
Computed tomographic scan showing consolidations in bilateral lower lungs (a), pulmonary embolism in the right pulmonary artery ((b), white arrowhead), and extensive DVT throughout inferior vena cava to left posterior tibial vein ((c)–(e), black arrowheads).

**Figure 2:**
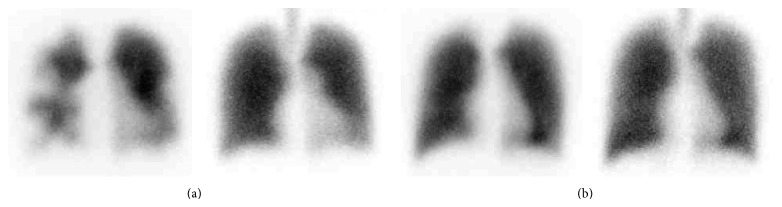
Ventilation and perfusion scan showing multiple areas of mismatch in bilateral lungs on admission (a) and resolved areas of mismatch after anticoagulant therapy (b).

On admission, physical examination showed decreased breathing sounds in her right back on auscultation and her left leg was swollen and painful. Her body temperature was 37.3°C, and she had a blood pressure of 104/62 mmHg. She had a regular heart rate of 92/min and a respiratory rate of 31/min with oxygen saturation of 98% in room air. Laboratory studies on admission revealed a white blood count of 10,220/*μ*L, hemoglobin level of 11.8 g/dL, and platelet count of 287 × 10^3^/*μ*L. The C-reactive protein level was 10.3 mg/dL. Liver function tests were normal. An electrocardiogram and echocardiogram did not reveal any abnormalities. Coagulation tests revealed normal prothrombin time, activated partial thromboplastin time, and fibrinogen, but a marked elevation of both FDP and D-dimer levels (113 *μ*g/mL and 46.8 *μ*g/mL, resp.). Chest radiography revealed consolidations in the bilateral lower lungs and a right pleural effusion. An enhanced CT scan showed a PE in the right pulmonary artery with bilateral consolidation in the lungs as well as extensive DVT throughout the inferior vena cava (IVC) to the left posterior tibial vein (Figure 1). A ventilation-perfusion scintigraphy confirmed multiple areas of mismatch compatible with a PE in both lungs (Figure 2(a)). She had no past or family history of thrombosis. Based on previous clinical symptoms and laboratory data, she was suspected to have (SLE-related) APS complicated with alveolar hemorrhage at the time of admission. She was treated with intravenous methylprednisolone pulse therapy (1 g/day for 3 days) followed by 1 mg/kg of oral prednisolone (PSL) in combination with oral tacrolimus (TAC) administration (3 mg/day). The day after admission, she had an IVC filter implanted and was given anticoagulation therapy with heparin and, a few days later, warfarin.

Compared with the previous results, her immunological tests in our hospital were negative for ANA and showed normal titers of aPL, including LAC (dRVVT), IgM aCL, and anti-*β*2 glycoprotein I (*β*2GPI) antibodies (Table 1). Other autoimmune markers for SLE were negative. To further explore thrombocytopenia, serological viral tests were performed. The results were positive for CMV-IgM and CMV-IgG and negative for hepatitis B surface antigen, hepatitis C, Epstein-Barr virus (EBV) viral capsid antigen IgM, EBV early antigen IgG, and parvovirus B19 IgM. To confirm whether or not it was an acute CMV infection, a PCR test for CMV (geni-Q) was performed using serum sample obtained on admission, and the tests revealed a positive result (200 copy/*μ*L). No pathological values for plasma anti-thrombin III, protein C, and S activity were found.

Taken together, she was thought to have developed DVT/PE and alveolar hemorrhage due to the transient appearance of aPL, that is, LAC, associated with acute CMV infection. Therefore, the PSL was tapered rapidly and TAC was stopped thereafter. The platelet count as well as the fibrinogen degradation product (FDP) and D-dimer values gradually returned to normal levels within three weeks. At the time of discharge, an antigenemia assay for CMV pp65 (C7-HRP) was negative. Seven months later, she was completely asymptomatic and her IVC filter was removed. Her CMV-IgM titer decreased with an elevation of her CMV-IgG titer (Table 2). Ten months later, a follow-up ventilation-perfusion scintigraphy (Figure 2(b)) and CT scan confirmed significant resolution of the previous PE and DVT. Finally, after one year, PSL and anticoagulant therapy were stopped, and she has been well without recurrence to date.

## 3. Discussion

Acute infections are known to be associated with a transient increased risk of venous thromboembolism, such as DVT and PE [3]. Thrombosis associated with CMV infection has been described, and the incidence of thrombosis among patients with acute CMV infection was estimated at 6.4% [4]. In contrast, a few prospective studies showed that the incidence of acute CMV infection among hospitalized patients with DVT and/or PE was 1.9–9.1% [5]. Early reports of thrombosis associated with acute CMV infection focused mainly on immunocompromised patients, such as HIV patients and transplant recipients. Recent reports, however, have also shed light on thrombotic events in immunocompetent patients with CMV infection. According to a recent literature search of 97 cases of thrombosis associated with acute CMV infection, two-thirds (64/97) of the cases were immunocompetent patients, and, among them, 13 cases (13/64; 20.3%) developed either transient or permanent APS syndrome [5].

Several mechanisms responsible for CMV-induced thrombosis have thus far been proposed. The virus can directly infect endothelial cells, causing vascular endothelial damage that in turn either activates coagulation factors or enhances platelet and leukocyte adhesion [6, 7]. CMV can also enhance coagulant properties by promoting the generation of thrombin or factor VIII and by inhibiting physiological anticoagulant mechanisms [8–10]. In addition, CMV may induce the production of aPL. This infection-associated aPL is generally considered not pathogenic because plasma protein *β*2GPI is not required as a cofactor to bind cardiolipins [11, 12]. However, among CMV-induced thrombosis associated with aPL, several reports described *β*2GPI-independent aPL [13–16], suggesting a pathogenic role.

To date, 6 cases of aPL-associated thrombosis (transient APS) following CMV infection have been described in the literature [13–18], and the features of these reported cases, including our case, are summarized in Table 3. Most cases were previously healthy individuals in their 20s and 30s, and all 6 cases had an elevated IgM/IgG aCL titer (5/6 cases had an elevated IgM aCL titer) but LAC was normal. Fever was a clinically universal feature in all cases, and other complications, such as arthralgia, diarrhea, and lymphadenopathy, were reported in two cases, respectively. Most cases had elevated liver enzymes, such as aspartate aminotransferase and alanine aminotransferase, and elevation of C-reactive protein was also common. All patients were treated with heparin and/or oral anticoagulants. Only one patient received antiviral agents, that is, ganciclovir and valganciclovir. Our patient received no antiviral therapy as well because she was originally an immunocompetent individual and made good progress after treatment. Additionally, serological monitoring of aPL showed that all 5 cases with an elevated IgM aCL titer had normal levels within 2–5 months, while one case with an elevated IgG aCL titer remained positive 6 months after disease onset.

While acute CMV infection was generally diagnosed by serologic testing, that is, positive CMV IgM, we identified our patient as a primary CMV infection by using a real-time PCR assay in addition to sequential serologic testing for CMV (Table 2). Indeed, the presence of CMV IgM may not be solely indicative of primary infection, since it is also produced during reactivation or reinfection of CMV. Therefore, the presence of CMV IgM should not be used by itself to diagnose primary CMV infection. In terms of thrombosis sites, a lower limb DVT and/or PE were most prevalent with acute CMV infection [5], which is consistent with most of the cases in Table 3, including our case.

How can CMV infection cause the production of aPL? Molecular mimicry has been proposed as the major mechanism for aPL synthesis generated by viral or other microbial infections [19, 20]. In fact, a previous study demonstrated that aPL induced by immunization of mice with a CMV peptide (TIFI) were pathogenic* in vivo* [21], suggesting a possible mechanism, that is, molecular mimicry, of induction of APS. In this study, some of the TIFI-induced aPL had LAC activity, which was also found in our case. In addition, another study showed that the levels of CMV-IgM in the aPL-positive patients were significantly increased compared with the levels in the control subjects [22], which also suggest a relationship between CMV infection and APS.

In our case, the patient had pulmonary alveolar hemorrhage. While thrombosis is the most common mechanism causing pulmonary complications in APS, alveolar hemorrhage is a rare manifestation of APS. Recently, however, there are a growing number of cases reporting APS-associated diffuse alveolar hemorrhage (DAH) [23, 24]. In these cases, like other causes of DAH, (aPL-induced) pulmonary capillaritis has been described as the underlying histopathology of this complication. It is possible that our patient had alveolar hemorrhage due to an immunological complication, such as capillaritis, because her radiographic-positive infiltrations were not all located near the thrombosis sites and improved rapidly with steroids before effective anticoagulant therapy.

The optimal treatment and management for patients with APS remain controversial and must be individualized according to the patient's clinical status and history of thrombotic events [25–27]. The finding that our patient appeared to develop a DVT/PE due to transient APS has an influence on both the type and duration of antithrombotic treatment. In such cases, lifelong anticoagulant therapy may not be necessary, and a meta-analysis of CMV-related thrombosis reported the treatment duration ranged between 20 days and 9 months [5]. In our case, we continued anticoagulation therapy for one year and stopped it once aPL and leg swelling disappeared, and then the DVT/PE improved.

## 4. Conclusion

Our case suggests an association between CMV infection and transient APS. To our knowledge, this is the first case of an immunocompetent patient with a primary CMV infection who developed a DVT and PE associated with a transient appearance of LAC. In light of previous reports along with our case showing that CMV-induced thrombosis in immunocompetent individuals is not rare, it is important to alert physicians to the association between CMV infection and thrombosis, in particular due to transient APS among healthy young individuals.

## Figures and Tables

**Table 1:** Clinical course and laboratory findings.

Clinical parameters (reference values)	Previous hospital	Our hospital		
	January 2013	January 2013^a^	February 2013	March 2013
ANA	1 : 320	(±)	(±)	(−)
Anti-ds DNA Ab	(−)	(−)	NT	NT
Anti-Sm Ab	(−)	(−)	NT	NT
IgG aCL (0–9 U/mL)	1 (−)	3 (−)	NT	NT
IgG anti-*β*2GPI (<3.5 U/mL)	<1.3 (−)	<1.3 (−)	NT	NT
LAC (dRVVT) (0–1.2)	1.24 (+)	1.17 (−)	0.98 (−)	0.99 (−)

aCL: anticardiolipin antibodies; ANA: antinuclear antibodies; *β*2GPI: *β*2 glycoprotein I; dRVVT: dilute Russell viper venom time; LAC: lupus anticoagulant; (−): negative; NT: not tested.

^a^Admission to our hospital.

**Table 2:** Results of serological, PCR, and antigenemia testing for CMV infection.

	Jan. 10 2013	Jan. 17 2013	Jan. 31 2013	Feb. 21 2013	Mar. 14 2013	Dec. 5 2013

CMV IgM (>0.80)	NT	1.84 (+)	2.50 (+)	4.33 (+)	2.83 (+)	0.91 (±)
CMV IgG (>2.0)	NT	5.9 (+)	11.8 (+)	10.7 (+)	9.7 (+)	14.4 (+)
CMV DNA (copy/*μ*L)	2 × 10^2^	NT	NT	NT	NT	NT
CMV antigenemia	NT	NT	(−)	NT	NT	NT

CMV: cytomegalovirus; (−): negative; NT: not tested.

**Table 3:** Characteristics of patients with CMV infection and vascular thrombosis associated with aPL.

Patient	Age/Sex	Diagnostic method of CMV infection	Site of thrombosis	Type of aPL	Reference
1	35/M	lgM+, viruria	Superior mesenteric vein, femoropopliteal vein	IgM aCL, IgG aCL (*β*2GPI-dependent)	[17]
2	32/F	lgM+	Iliac vein	IgM aCL	[13]
3	79/F	lgM+, antigenemia+	Pulmonary artery	IgG aCL	[14]
4	30/M	lgM+, antigenemia+	Portal vein	IgM aCL	[15]
5	24/F	lgM+ (antigenemia−)	Superior mesenteric vein, pulmonary artery	IgM aCL	[16]
6	30/F	lgG+, mononucleosis-like syndrome	Middle cerebral artery	IgM aCL, IgG aCL, IgM anti-*β*2GPI, IgG anti-*β*2GPI	[18]
Present case	19/F	lgM+, real-time PCR	Inferior vena cava, posterior tibial vein, pulmonary artery	LAC	—

aCL: anticardiolipin antibodies; aPL: antiphospholipid antibodies; *β*2GPI: *β*2 glycoprotein I; CMV: cytomegalovirus; LAC: lupus anticoagulant.
